# Stroke in women: experience in a developing country

**DOI:** 10.1186/s12883-023-03314-3

**Published:** 2023-07-17

**Authors:** Nevine El Nahas, Hany Aref, Fatma Fathalla Kenawy, Shady Georgy, Eman Mones Abushady, Noha Lotfy Dawood, Sara Hamdy, Nourhan Abdelmohsen, Yasmine Hassan Abdel Hamid, Tamer Roushdy, Hossam Shokri

**Affiliations:** Neurology Department, Faculty of Medicine, Ain Shams University, Cairo, PO 11591 Egypt

**Keywords:** Gender, Women, Stroke, Thrombolysis

## Abstract

**Background:**

Several studies have addressed gender differences in stroke. Yet, results are diverse, and research is still required in different populations. So, this study investigates variation in stroke according to gender in a developing country.

**Methods:**

This is a registry-based, retrospective observational cross-sectional study comparing men and women as regards age, risk factors, stroke severity, quality of services, and stroke outcome.

**Results:**

Data analyzed comprised 4620 patients. It was found that men outnumbered women, while women had an older age, more prevalence of hypertension and atrial fibrillation, with severer strokes and worse outcomes. However, there was no gender difference in promptness nor frequency of administration of revascularization therapies.

**Conclusion:**

Despite the gender difference in risk factors and stroke severity, we could not detect any significant disparity in acute stroke services provided to either gender. Among age categories in women, we identified differences in acute ischemic stroke subtypes, and acute management in favor of older age.

## Introduction

Stroke ranks among the commonest causes of disability worldwide thus lack of understanding of gender differences can lead to mismanagement in acute and chronic stroke settings [1]. Multiple studies addressing stroke in women have been published and have inspired the medical community. Despite that, studies of gender-specific risk factors in stroke are controversial among different countries and ethnic groups [2, 3].

In South America [4] and South Asia [5] stroke prevalence was higher in men above 65, but lately, according to the global burden of disease report for years 1990–2019, the age-standardized incidence rates did not differ between both genders [6]. Additionally, some but not all studies found hypertension to be more prevalent in women with stroke [7] yet others related it to ethnic groups [8].

Many studies stated that diabetes is a stronger risk factor in women than men [7] while others found no difference in glycated hemoglobin between both genders [9].

Similarly, conflicting results were reported for dyslipidemia [10].

On the other hand, several studies attributed stroke risk in women to gender-related factors such as oral contraceptive intake, pregnancy, and menopause [11].

Moreover, reporting on stroke severity varied among studies possibly due to variability of measuring scales [12]. Some reported severer strokes and bad outcomes in women, [13] while others found no such gender difference [14]. A study of 2534 patients in Poland [15], a meta-analysis [16], and a study in Sub-Saharan Africa [17], all pointed to higher mortality rates in women. In contradistinction, a South American study showed the opposite [18].

And even more important is the conflicting data on gender differences in time delay to acute ischemic stroke (AIS) treatment (onset to door and door to needle times) and consequently the lack of reports on the quality of services and patient outcome [19]. Also, the rates of administration of intravenous thrombolysis (IVT) and mechanical thrombectomy (MT) in AIS are inconsistent [19–22]. The reported lower rates of revascularization therapies in women have been attributed to an atypical presentation that leads to treatment delays [23].

It is worth mentioning that despite the seemingly small difference in quality of service illustrated in some studies, this might in effect deprive women of the only medication approved for AIS therapy and can result in poorer outcomes in women.

Accordingly, data from different countries with different ethnicities are still required to verify these controversies. Thus, the objectives of our study are to investigate gender differences as regards age, stroke risk factors, stroke severity, and outcome, quality of services namely time to treatment, and administration of revascularization therapies in a cohort derived from the stroke unit registry (The Safe Implementation of Treatments in Stroke, SITS) of a developing country.

## Methods

This is an observational, cross-sectional, retrospective registry-based study using data from the stroke unit registry (The Safe Implementation of Treatments in Stroke, SITS) of a tertiary care university hospital. The study was conducted after the approval of the IRB at the faculty of medicine, at Ain Shams University. All experimental protocols were approved by the same committee. This hospital serves a catchment area of 6 million people. The stroke registry comprises patients presenting with acute stroke to our stroke centers from April 2015 till October 2021. It contains data regarding stroke patients’ demographics, risk factors, stroke severity, and outcome as well as details of stroke services provided concerning onset to the door and door to needle/groin times.

Inclusion criteria comprised patients who are above 18 years of age and included both genders presenting with any type of acute stroke. Exclusion criteria were age below 18 years and final diagnosis other than stroke such as stroke mimics. Informed consent was obtained from all subjects before admission to the stroke unit as all the procedures performed were part of the standard stroke unit care. The whole group was categorized according to age into 3 groups: (18–45 years), (46–60 years) and (> 60 years) which were labeled in women as childbearing (CB), menopause (M), and post-menopause (PM), respectively. This categorization is based on different risk exposures in women’s life according to age group. In the age group (18–45 years), a woman is at risk of oral contraceptive intake, pregnancy, and puerperium, in the age group (46–60 years) she has passed the reproductive period and is still protected by the endogenous estrogen. Then in the group above 60, women are more at of risk being deprived of endogenous estrogen and are liable to receive hormone replacement therapy, in addition to a higher prevalence of classic vascular risk factors.

Women were compared then to men as regards risk factors, stroke severity measure by the National Institutes of Health Stroke Scale (NIHSS), and outcome where favorable outcome refers to (≤ 2 on the Modified Rankin Scale; mRS), type of stroke, acute management with time factors related to it and age category.

Women were further compared within age categories for the type of stroke, acute intervention, stroke severity, and outcome, and time factors related to acute management.

### Statistics

Statistical analysis was done using SPSS version 19th version Statistics (SPSS Inc., Chicago). The Shapiro-Wilks test was used to test for the normality of continuous data distribution. Mean and standard deviation was used for normally distributed data, while median and interquartile range (IQR) were used for skewed data. Categorical data were presented as frequencies. Mann-Whitney Test and Kruskal-Wallis test are used to compare not normally distributed continuous variables with nominal independent variables. The chi-square test was used for comparison of nominal data.

## Results

### Risk factors and clinical data

The total number of patients was 4620, of whom 1813 (39.2%) were women. Women were significantly older than men (p = < 0.001), showing more prevalence of hypertension, atrial fibrillation (AF) (p = < 0.001, each), and higher serum cholesterol (p = 0.001), with less prevalence of smoking (p = < 0.001) and of other vascular diseases (p = 0.005). They had significantly severer strokes verified by a higher NIHSS on presentation, and at discharge (p = 0.001), and the percentage of women with favorable outcomes by mRS was significantly less than men (p = < 0.001). No statistically significant difference was detected between genders for the type of stroke whether ischemic, hemorrhagic, or transient ischemic attacks (TIA). However, subarachnoid hemorrhage was higher in women (p = 0.003). There was no difference in most modalities of acute treatment provided to either gender, except that women were more likely to receive bridging therapy (IVT followed by MT) (p = 0.002). Also, all the time-related factors showed non-significant differences among genders; time from onset of stroke to door (p = 0.9), onset to needle (p = 0.3), and door to needle times (p = 0.2) (Table 1).

**Table 1:** Comparison between both genders regarding stroke demographics

	Women (n = 1813)	Men(n = 2807)	p-value
Age^*^	65 (55–71)	62 (54–69)	**< 0.001**
Atrial Fibrillation (admission/past history)	19.8%	8.7%	**< 0.001**
Hypertension (admission/past history)	69.6%	60.6%	**< 0.001**
Diabetes (admission/past history)	47.9%	46.4%	0.4
Hyperlipidemia (admission/past history)	9.7%	9.5%	0.9
Current Smoker	2.2%	18.8%	**< 0.001**
PreviousStrokeEarlierThan3Months	9.7%	10.4%	0.5
Previous TIA	1.4%	1.5%	0.8
Congestive Heart Failure	2.1%	2.6%	0.4
Vascular Disease	10.1%	13.1%	**0.005**
Glucose (mg/dl) *	160 (122–212)	160 (121–215)	0.811
Cholesterol (mg/dl) *	192 (154–229)	183 (146–219)	**0.001**
NIHSS admission*	7 (4–12)	6 (3–10)	**< 0.001**
NIHSS discharge*	4 (2–7)	3 (2–6)	**< 0.001**
*Type of stroke*:			
Ischemic stroke	85.6%	86.6%	0.3
Hemorrhagic Stroke	7.1%	7.7%	0.4
TIA	2.4%	2.4%	1
Subarachnoid hemorrhage	5%	3.3%	**0.003**
*Acute Intervention/treatment*			
Conservative	82.8%	82.1%	0.5
IVT	15.2%	16.7%	0.1
MT	1.2%	1%	0.5
IVT, MT	0.8%	0.2%	**0.002**
Onset to door (minutes)*	492 (240–1440)	534 (240–1400)	0.9
Onset to needle (minutes)*	160 (120–210)	179 (120–210)	0.3
Door to needle (minutes)*	40 (30–60)	45 (30–60)	0.2
mRS* 3 months (favorable outcome)	47.3%	56.1%	**< 0.001**

* Median (IQR), TIA: transient ischemic attack, NIHSS: National Institutes of Health Stroke Scale, IVT: intravenous thrombolysis, MT: mechanical thrombectomy, mRS: Modified Rankin Scale favorable outcome: (≤ 2)

### Age categories of the studied population

**Fig. 1:**
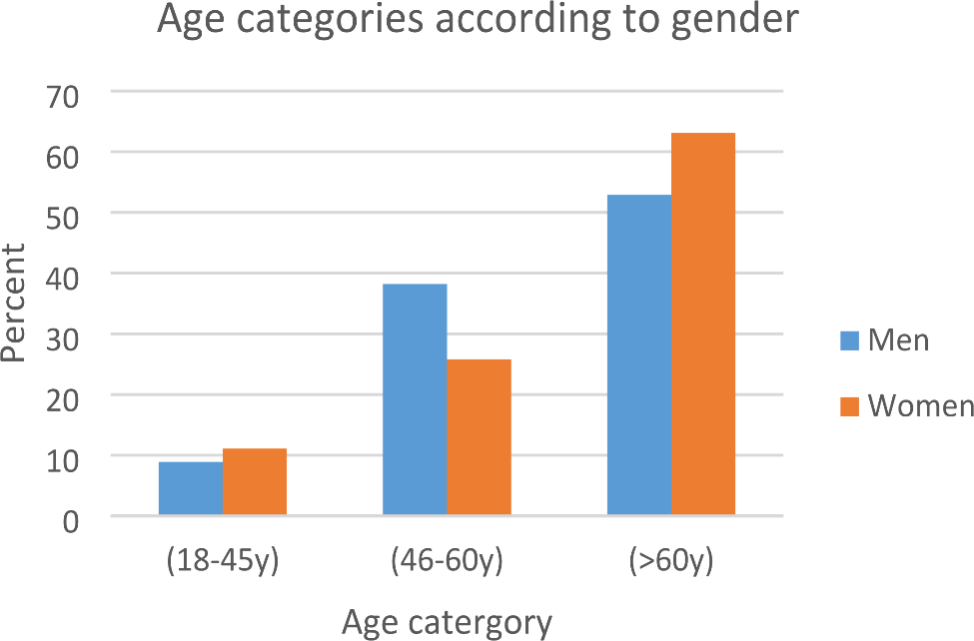
Age categories according to gender

Among the whole sample, the older age group (> 60 years) represented the highest percentage (56.9%) of all patients and the younger group (18–45 years old) represent the least percentage (9.8%). Men were predominant in the middle age group (46–60 years) (p = 0.0001), while women predominated in the younger and older groups (childbearing and post-menopause groups), being significantly more in the post-menopause group (> 60 years) (p = 0.0001) (Table 2; Fig. 1).

**Table 2:** Age categories of the studied population

	The Whole Sample	Men	Women		p-value
(18-45y)	9.8% (450)	8.9% (249)	Childbearing	11.1% (201)	0.2
(46-60y)	33.4% (1538)	38.2% (1072)	Menopause	25.8% (466)	**0.0001**
(> 60y)	56.9% (2622)	52.9% (1483)	Post-menopause	63.1% (1139)	**0.0001**

### Types of strokes in different age categories among women

Ischemic stroke showed a significant difference among the three groups with an increased frequency with higher age (p = < 0.0001). On the other hand, hemorrhagic stroke showed an opposite trend i.e., decreased frequency with increasing age (p = < 0.001). Also, subarachnoid hemorrhage showed decreased frequency with increasing age and was significantly less in the PM group than in the M or CB groups. TIA was not different among groups (Table 3).

**Fig. 2:**
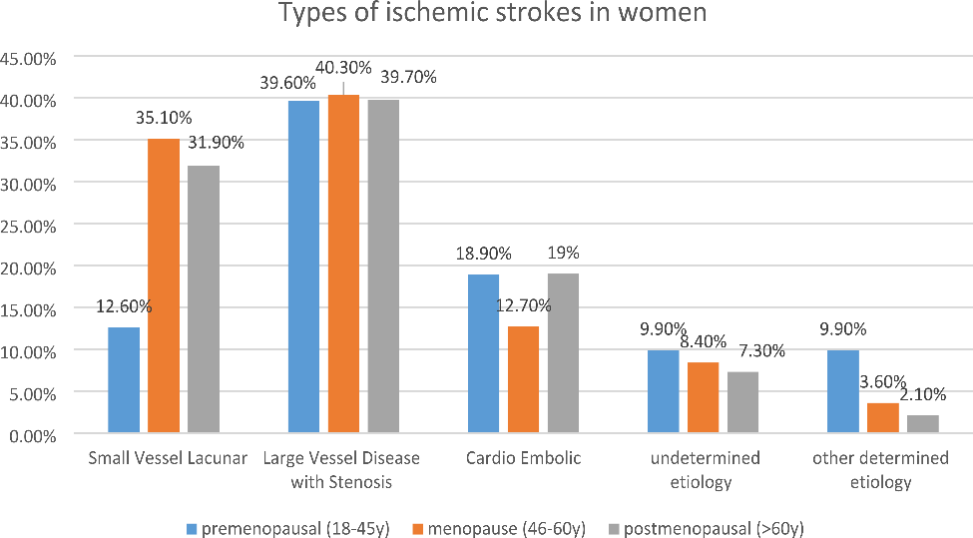
Types of ischemic stroke among women

Regarding ischemic stroke subtypes, it was found that small vessel disease (SVD) was significantly higher in the older age groups M and PM compared to CB (p = < 0.0001), and also higher in PM than M (p = 0.03). Cardioembolic stroke was more in the age extremes (CB, PM) compared to the M group (p = 0.0009 and < 0.0001 respectively). Other determined etiology was significantly more in the younger age group (CB) followed by the M group compared to the PM group (p = < 0.001 and 0.003, respectively) (Table 3; Fig. 2).

Comparison of the type of acute intervention revealed that CB and M were more likely to receive conservative therapy compared to PM (p = < 0.0001 and 0.0002, respectively), while PM women were more likely to receive IVT compared to M and CB (p = < 0.0001 and 0.0004 respectively), whereas MT did not differ among age groups.

The number of patients with favorable outcomes on discharge and at 3 months follow-up showed a significant decrease with older age (p = 0.002 and 0.001 respectively) (Table 3).

**Table 3:** Comparison of different age categories among women

	CB	M	PM	p-value
Age Frequency	(18-45y) N = 450	(46-60y) N = 1538	(> 60y) N = 2622	
*Type of stroke, %*				
Ischemic stroke	68.1	79.4	91.3	**< 0.0001**
Hemorrhagic Stroke	16.7	8.6	4.7	
TIA	2.9	2.2	2.4	
Subarachnoid Hemorrhage	12.3	9.8	1.5	
*Post-hoc*	p-value			
Ischemic stroke	**CB vs M**	**M vs PM**	**CB vs PM**	
Hemorrhagic Stroke	**0.001**	**0.0001**	**0.0001**	
TIA	**0.0001**	**0.0001**	**0.0001**	
Subarachnoid Hemorrhage	0.3 0.1	0.6 **0.0001**	0.5 **0.0001**	
*Type Of ischemic stroke*				
Small Vessel Lacunar	12.6	35.1	31.9	**< 0.0001**
Large Vessel Disease with	39.6	40.3	39.7	
Stenosis				
Cardio Embolic	18.9	12.7	19	
Undetermined etiology	9.9	8.4	7.3	
Other determined etiology	9.9	3.6	2.1	
*Post-hoc*	p-value			
	**CB vs M**	**M vs PM**	**CB vs PM**	
Small Vessel Lacunar	**0.0001**		**0.0001**	
Large Vessel Disease with	0.7	**0.03**	0.9	
Stenosis		0.7		
Cardio-embolic	**0.0009**	**0.0001**	0.9	
Undetermined etiology	0.3	0.1	0.05	
Other determined etiology	**0.0001**	**0.003**	**0.0001**	
*Acute Intervention treatment*				
Conservative	88.1	85.6	80.9	**0.05**
IVT	10.4	12	17.1	
MT	1	1.1	1.3	
IVT and MT	0.5	1.3	0.6	
Post-hoc	p-value			
	**CB vs M**	**M vs PM**	**CB vs PM**	
Conservative	0.1	**0.0001**	**0.0002**	
IVT	0.3	**0.0001**	**0.0004**	
MT	0.8	0.5	0.5	
IVT and MT	0.1	**0.01**	0.7	
mRS* discharge favorable outcome	54.2	38.4	31.9	**0.002**
Post-hoc	**CB vs M**	**M vs PM**	**CB vs PM**	
p-value	**0.0001**	**0.0001**	**0.0001**	
mRS* 3 months favorable outcome	59.6	51.5	43.6	**< 0.001**
Post-hoc	**CB vs M**	**M vs PM**	**CB vs PM**	
p-value	**0.002**	**0.0001**	**0.0001**	

CB: childbearing (18-45y), M: menopause (46-60y), PM: postmenopausal (> 60y), TIA: transient ischemic attacks, IVT: intravenous thrombolysis, MT: mechanical thrombectomy, mRS: Modified Rankin Scale favorable outcome: (≤ 2)

### Stroke severity and time factors in the women group

Stroke severity measured by NIHSS demonstrated a significant increase associated with increasing age as shown in Table 4. While onset-to-door time did not show a consistent trend related to age. The m group had a significantly longer time than the PM group, and so did the CB group, with no significant difference between CB and M groups. Onset-to-needle and door-to-needle times did not differ significantly among age groups (Table 4).

**Table 4:** Stroke severity and time factors in the women group

	CB	M	PM	p-value
**NIHSS admission**	6 (2–10)	7 (3–12)	7 (4–12)	< 0.001
**Post-hoc**	p-value			
	CB vs. M	M vs. PM	CB vs. PM	
	0.01	0.03	< 0.001	
Onset to door (minutes)*	540 (300–1560)	600 (337–1440)	480 (210–1380)	0.005
**Post-hoc**	**p-value**			
	CB vs. M	M vs. PM	CB vs. PM	
	0.7	0.006	0.02	
Onset to needle (minutes)*	180 (150–206)	150 (120–200)	165 (125–210)	0.325
Door to needle (minutes)*	30 (30–45)	42 (30–60)	40 (30–60)	0.761

*Median (IQR), CB: childbearing (18-45y), M: menopause (46-60y), PM: postmenopausal (> 60y)

## Discussion

In this study, we investigated a cohort of stroke patients derived from the SITS data of a developing country to identify gender differences in stroke as regards vascular risk factors, stroke severity, and outcome as well as the quality of AIS services provided to each gender.

We found that women were older than men, a finding previously reported in an Indian population [24] and a German cohort [25]. This age difference was significant in the older age group > 60 years, which agrees with *Danesi et al., 2013* in Nigeria, [26]. Rural Tanzania [27] and with a Danish population [28]. The higher age of women presenting with stroke is possibly due to the longer life expectancy [29].

In our cohort, the total number of men exceeded that of women, which agrees with some past studies [30] but differs from Corbière et al (2021) who stated that in Arab countries the incidence of stroke was more in women [31].

Similar to previous studies, [31, 32] women outnumbered men in two age extremes. The younger childbearing group is more liable to migraine, oral contraceptive intake, and pregnancy-related risk of thrombosis in the peripartum period [33–35]. In the postmenopausal group, loss of the protective endogenous estrogen seems to raise the risk for stroke, as shown in experimental animal models [36].

Hypertension and AF were significantly more in women while smoking and vascular diseases were more in men, which agrees with several other studies [37–40]. We concurred with *Denish et al. 2015* [41] who found no gender disparities for diabetes or dyslipidemia yet studies for a Chinese cohort [42] and some Western studies reported that women were more liable for diabetes and dyslipidemia [43], and women with diabetes had a worse prognosis [44]. A similar distribution of risk factors is observed in the rare condition of transient global amnesia (TGA), which is considered in itself a risk factor for stroke [45].

On the other hand, women presented with a severer stroke in addition to having a less favorable outcome at 3 months. The poor outcomes can be attributed to a higher prevalence of AF and hypertension as previously described [46, 47]. The seriousness of AF as a risk factor for women has inferred the inclusion of the female gender with AF in the CHA2DS2-VASc stroke risk [48]. The poorer stroke outcome in women cannot be explained by any variance in the type of acute management since even when IVT was administered to women, still the functional outcome was worse [49, 50].

Unlike previous studies, [51] we did not demonstrate any gender differences as regards the quality of services. In our cohort, IVT and MT were equally administered to both genders similar to Weber et al. 2019 [19] and other centers in USA and Austria [52, 53]. The only difference detected was that women were more liable to be treated by bridging thrombolysis (IVT followed by MT), which might denote more proximal vascular occlusion in women.

Also, we did not identify any significant difference concerning time factors related to IVT including onset to treatment times. There was rather a trend towards shorter durations in women. This is discordant with other studies denoting that women were less likely to receive thrombolytic therapy and this was partly attributed to less adherence to management guidelines [51, 54, 55]. Besides, a meta-analysis of 17 studies, with over a million stroke patients, indicated that treatment with IVT was less likely in women than men [56]. Furthermore, several scholars from Hong Kong, Australia, Puerto Rico, the USA, and others reported that onset to treatment times was delayed in women [13, 40, 57–60].

The reduced revascularization treatment rates among women have sometimes been ascribed to severer stroke at onset, older age, stroke mimics among young women [61], as well as lack of stroke therapy guidelines for women as they tend to be under-represented in RCTs [62].

It was also observed that the type of treatment differed according to age category so that the CB age group was more likely to receive conservative treatment, possibly because stroke severity was less, or they were more liable to be misdiagnosed as stroke mimics [63]. The postmenopausal group had a shorter onset to door time and were thus likely to receive revascularization therapy.

Types of strokes were similar to other studies, subarachnoid hemorrhage being significantly more in women, [64, 65] ischemic stroke was commoner in the post-menopausal category, while intracerebral and subarachnoid hemorrhage were more prevalent in the CB age group.

As for subtypes of AIS according to TOAST classification, the CB age group showed a preponderance of cardioembolic and undetermined strokes however, they were similarly inflicted by large vessel disease as the older groups. This reflects the rising prevalence of traditional risk factors such as hypertension, diabetes, and dyslipidemia among young stroke patients [66].

On the other hand, SVD was significantly more in the M and PM groups while cardio embolism displayed a second surge in the M group possibly due to AF [67].

Compared to our population, Giralt et al. 2011 [43] and several others [54, 68, 69] found that cardioembolic stroke was commoner in women and that athero-thrombotic stroke was commoner in men. And this is comprehensible given the higher prevalence of AF in women. Nevertheless, Smith et al. 2005 did not find such a discrepancy [70].

## Conclusion

The gender differences detected may be attributable to biological differences and lifestyle, while stroke services provided in the hospital were fairly equal for both genders. This implies the need for awareness programs to control risk factors and implement a healthy lifestyle.

### Limitations

The main limitation of our study is the retrospective design where data may be subjected to sampling bias. Also, no comprehensive follow-up was performed as many patients were interviewed by phone calls.

### Recommendations

With women having a longer life expectancy and more prevalence of hypertension and AF [71], they are speculated to have a greater lifetime risk for stroke [72, 73]. Accordingly, stroke services should be equally provided worldwide, regardless of gender, age, or social status.

## Data Availability

The datasets generated during the current study are not publicly available but are available from the corresponding author on reasonable request.
